# Plasma 25-hydroxyvitamin D levels, vitamin D intake, and pancreatic cancer risk or mortality: a meta-analysis

**DOI:** 10.18632/oncotarget.18888

**Published:** 2017-06-29

**Authors:** Xi Zhang, Xuan-Zhang Huang, Wen-Jun Chen, Jian Wu, You Chen, Cong-Cong Wu, Zhen-Ning Wang

**Affiliations:** ^1^ Department of Chemotherapy and Radiotherapy, The Second Affiliated Hospital and Yuying Children's Hospital of Wenzhou Medical University, Lucheng, Wenzhou 325027, P.R. China; ^2^ Department of Pediatric Dentistry, The Wenzhou Dental Hospital, Lucheng, Wenzhou 325027, P.R. China; ^3^ Department of Surgical Oncology and General Surgery, The First Hospital of China Medical University, Heping, Shenyang 110001, P.R. China

**Keywords:** vitamin D, 25-hydroxyvitamin D, pancreatic cancer, risk, mortality

## Abstract

**Background:**

The associations between vitamin D status, including plasma 25-hydroxyvitamin D [25(OH)D] levels and vitamin D intake, and pancreatic cancer risk and mortality are inconsistent. The aims of this study are to evaluate the antitumor and therapeutic effects of vitamin D status for pancreatic cancer patients.

**Methods:**

A literature search for relevant studies was conducted using PubMed and Embase databases. Risk ratio (RR), hazard ratio (HR), and 95% confidence interval (CI) were used as the effect measures. All statistical analyses were performed using Stata software 12.0.

**Results:**

Our results indicated that high plasma 25(OH)D levels were inversely associated with pancreatic cancer mortality without significant heterogeneity (HR=0.81, 95% CI=0.68–0.96). However, high plasma 25(OH)D levels could not reduce pancreatic cancer risk (RR=1.02, 95% CI=0.66–1.57). Moreover, vitamin D intake was also not associated with pancreatic cancer risk (RR=1.11, 95% CI=0.67–1.86)

**Conclusions:**

Our results indicate that high plasma 25(OH)D levels were significantly associated with improved survival in pancreatic cancer patients. However, there were no significant associations between vitamin D intake or plasma 25(OH)D levels and pancreatic cancer risk.

## INTRODUCTION

Pancreatic cancer is a highly fatal malignancy for which the incidence is increasing and mortality closely parallels the incidence [1, 2]. Surgical resection is regarded as the only potentially curative treatment for pancreatic cancer [3]. However, more than half of patients are not feasible for surgical resection at first diagnosis and there are no other effective treatments [4]. Advances in the improved survival are slow for pancreatic cancer, for which the 5-year relative survival is about 8% [2]. Thus, it is important to identify modifiable risk factors for pancreatic cancer incidence and mortality.

Vitamin D is a fat-soluble vitamin that correlates with calcium, phosphate, and bone metabolism [5]. Plasma 25-hydroxyvitamin D [25(OH)D], the precursor of the physiologically active form of vitamin D, is considered the best biomarker of vitamin D status because plasma 25(OH)D levels reflects both vitamin D intake from diet and synthesis from ultraviolet-B (UVB) exposure [6, 7]. In recent years, the role of vitamin D and its analogues in carcinogenesis has drawn more and more attention. Indeed, several studies have shown that vitamin D intake or plasma 25(OH)D levels are associated with the risk and mortality of breast cancer and colorectal cancer [8–11]. And for pancreatic cancer, experimental evidence demonstrates that vitamin D and its analogues may inhibit pancreatic cancer cell proliferation, induce differentiation, and promote apoptosis [12–16]. However, for clinical studies, the results on the association between vitamin D status, including vitamin D intake or plasma 25(OH)D levels, and pancreatic cancer risk and mortality are still inconsistent.

Thus, the purposes of this study are to assess whether vitamin D intake or plasma 25(OH)D levels is associated with pancreatic cancer risk and mortality through meta-analysis approach.

## RESULTS

### Study selection

**Figure 1:**
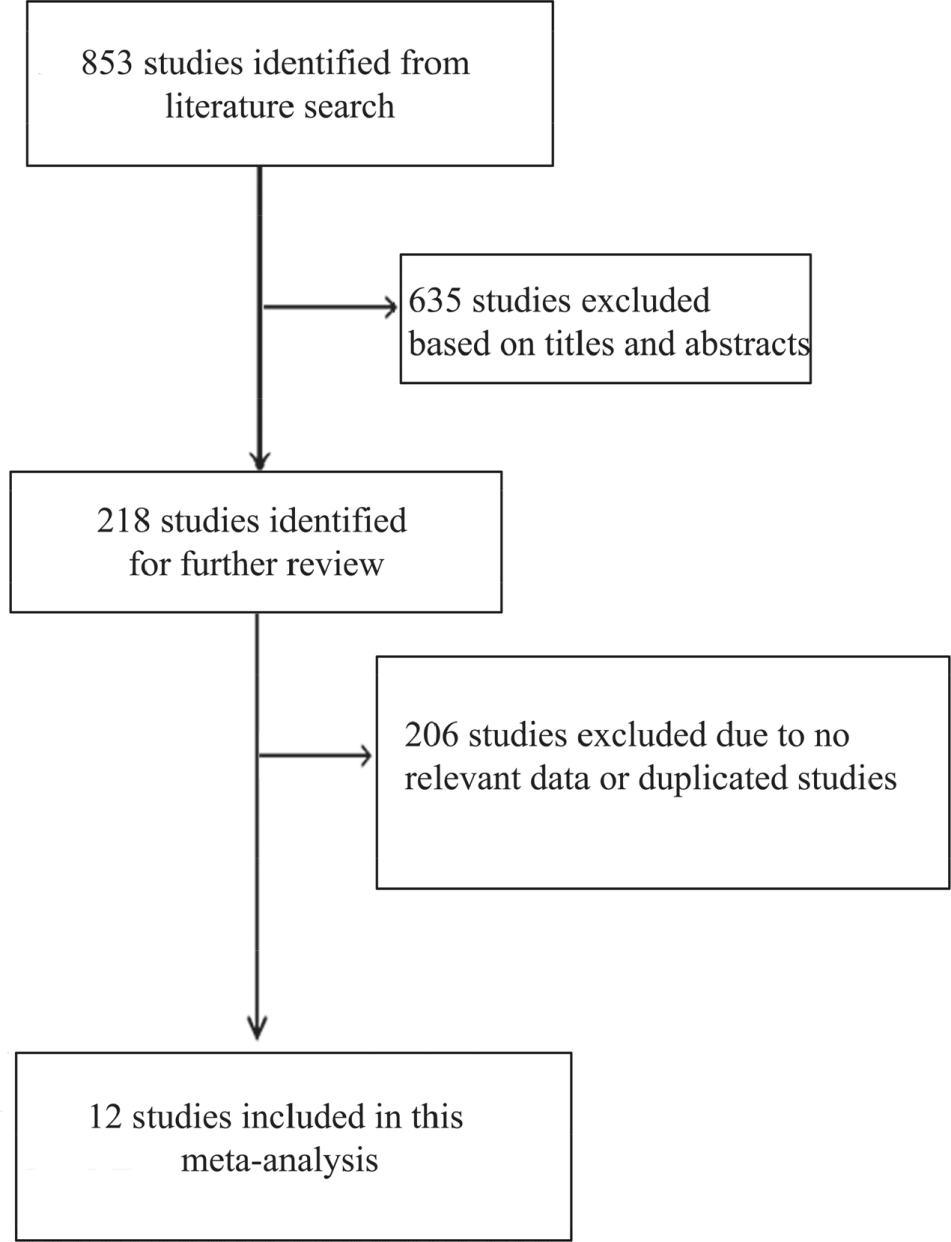
Literature search and study selection

There were 853 studies identified from literature search, and 635 studies were excluded after reviewing titles and abstracts. The remaining 218 studies were evaluated based on full-text review, and 206 studies were excluded based on eligible criteria. Finally, twelve studies were included (Figure 1) [17–28].

### Study characteristics

Five studies on pancreatic cancer mortality in relation to plasma 25(OH)D levels were published between 2013 and 2016, and involved a total of 1613 patients [19, 21, 22, 25, 28] (Table 1). Of these five studies, four were conducted in the United States and one in Germany. Five studies on pancreatic cancer risk in relation to plasma 25(OH)D levels were published between 2010 and 2015, and involved a total of 17385 patients [17, 18, 23, 24, 27] (Table 2). Of these five studies, four were conducted in the United States and one in Denmark. Two studies on pancreatic cancer risk in relation to vitamin D intake were published between 2014 and 2015, and were conducted in the United States, involving a total of 874170 patients [20, 26] (Table 3). The baseline characteristics and quality of the included studies were shown in Tables 1–3.

**Table 1:** The main characteristics of included studies on plasma 25(OH)D levels and pancreatic cancer mortality

Article	Country & year	Study type	Study name	Sample (M/F)	Age (years)	Tumor feature	BMI	Baseline 25(OH)D (ng/mL)	Study quality
Yuan	USA 2016	Cohort	Health Professionals Follow-up Study, Nurses’ Health Study, Physicians’ Health Study, Women's Health Initiative and Women's Health Study	493(148/345)	Mean:71.2; SD:8.3	Localized:65;locally advanced:120;M1:220;unknow:88	BMI<25.0:216;BMI:25.0-29.9:182;BMI≥30.0:95	Mean:24.6; SD:9.84	7
McGovern	USA 2016	Cohort	Five Cancer Treatment Centers of America hospitals	627(342/285)	Mean:57;SD:7.7; range:31-82	I-II:83;III:86;IV:454	BMI<18.5:26;BMI:18.5-24.9:264;BMI:25-29.9:207;BMI≥30:130	Mean:male:28,SD:14;female:30,SD:17	6
Haas	Germany 2015	Cohort	German Pancreatic Cancer Center (Comprehensive Cancer Center)	59(33/26)	Median:69; range:39-84	Locally advanced:11;M1:48; previous surgery:16	NR	Median:19.9;range:10.0-42.0:	6
Van Loon	USA 2014	Cohort	Cancer and Leukemia Group B 80303	256(136/120)	Median:64; range:35-84	Locally advanced:217;M1:39	BMI<25:126;BMI≥25:130	Median:21.7;range:4-77:	7
Cho	USA 2013	Cohort	Siteman Cancer Center in St. Louis	178(96/82)	<50y:17; ≥50y:161	I+II:64;III+IV:114	BMI <25:68;BMI≥25:110	NR	7

25(OH)D: 25-hydroxyvitamin D; BMI: body mass index; M1: metastatic disease; M/F: male/female; NR: not reported; SD: standard deviation; USA: the United States of America.

**Table 2:** The main characteristics of included studies on plasma 25(OH)D levels and pancreatic cancer risk

Article	Country	Study design	Sample (M/F)	Age (years)	Follow up (years)	Baseline 25(OH)D (ng/mL)	BMI	Adjusted variables	Study quality
Piper 2015	USA	Nested CCS	882(549/333)	Median: 65; IDR:57-71	Follow-up to 15.1	Case:24.36(IDR11.28-37.32);conrol:25.28 (14.16-36.28)	BMI<25:306;BMI:25-30:395;BMI≥30:181	Age, race-ethnicity, sex, and date of blood draw, smoking status and diabetes	7
Ananthakris-hnan 2014	USA	Cohorts	2809 (1097/1712)	Median:46; IQR:32-60	Median:11(IQR5-18)	Median:26;IQR:17-35	NR	Age, sex, race, measurement season, follow-up duration, immunosuppression use and IBD type	6
Afzal 2013	Denmark	Cohorts	9791 (4358/5431)	<5ng/ml:59(IQR50-65); 5-9.9:58(IQR49-65);10-19.9:58 (IQR48-64);≥20:57(IQR47-64)	Median:21;range:0.01-28	Median:16.4	<5ng/ml:25 (22-29);5-9.9:26 (23-29);10-19.9:25 (23-28);≥20:24 (22-27)	Age, sex, pack-years, BMI, alcohol consumption, leisure time, physical activity and education duration	7
Wolpin 2012	USA	Nested CCS	1618 (518/1100)	Mean:62.5;SD:8.5	Median:14.3	Mean:25.8; SD:9.28	Mean:25.9; SD:4.5	Age, cohort, BMI, smoking status, history of diabetes mellitus, multivitamin use and month of blood draw.	7
Stolzenberg-Solomon 2010	USA	Nested CCS	2285 (1521/764)	Median:case:62(IQR56-68);control:62(IQR57-67)	Median:6.5;range:0-30.6	Range:case:0.8-62.4;control:1.04-50.88	BMI<25:876;BMI:25-30:935;BMI≥30:356	Age, race/ethnicity, sex, cohort, date of blood draw, BMI, smoking and diabetes status.	7

25(OH)D: 25-hydroxyvitamin D; BMI: body mass index; CCS: case-control study; IDR: interdecile range; IQR: interquartile range; M/F: male/female; NR: not reported; SD: standard deviation; USA: the United States of America.

**Table 3:** The main characteristics of included studies on vitamin D intake and pancreatic cancer risk

Article	Country	Year	Study design	Age (years)	Smoking	BMI	Ethnicity (white/black)	Sample (M/F)	Adjusted variables	Study quality
Waterhouse	USA	2015	Case-control study	Median:65	Ever:6525; never:4948	BMI<25:4491;BMI:25-30:4148; BMI:30-35:1422; BMI≥35:585	9159/408	11490(6165/5325)	Age, sex, smoking status, total daily energy intake and other cohort-specific confounders (alcohol intake, BMI, history of diabetes, history of pancreatitis, family history of pancreatic cancer, education, race, total calcium intake, dietary calcium intake, total retinol intake, dietary retinol intake, total vitamin A intake, study centre and extent of proxy use)	7
Genkinger	USA	2014	Cohorts	Range:15-107;most:≥40	NR	NR	NR	862680(319732/542948)	Smoking habits, personal history of diabetes, alcohol intake, BMI, energy intake, age in years and year of questionnaire return	6

BMI: body mass index; M/F: male/female; NR: not reported; USA: the United States of America.

### Association between plasma 25(OH)D levels and pancreatic cancer mortality

**Figure 2:**
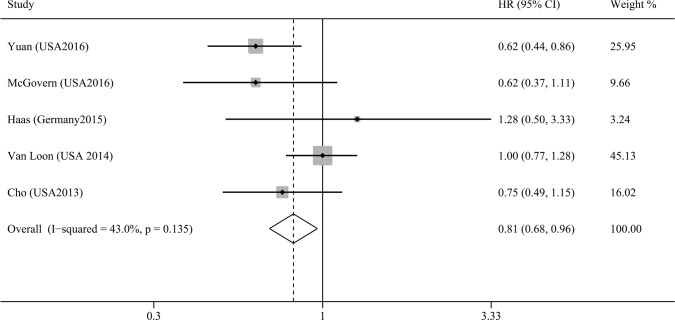
Meta-analysis of the association between plasma 25(OH)D levels and pancreatic cancer mortality

**Figure 3:**
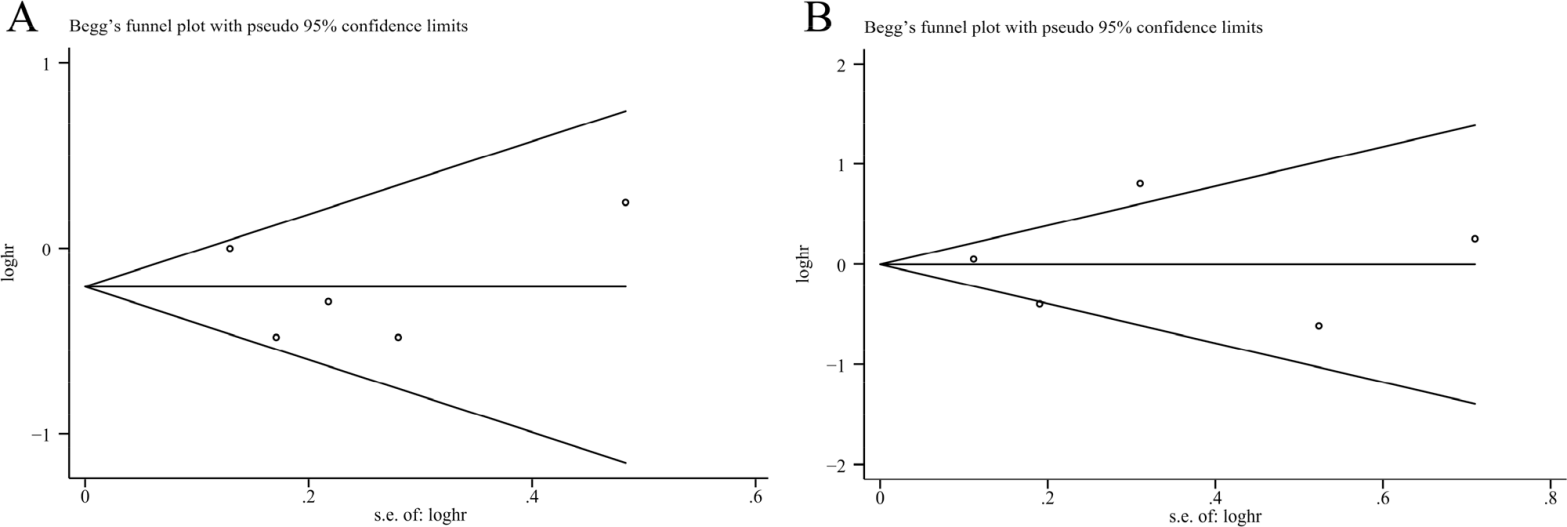
Funnel plots assessing publication bias for plasma 25(OH)D levels and pancreatic cancer mortality **(A)** and pancreatic cancer risk **(B)**.

Five studies assessed the association between plasma 25(OH)D levels and pancreatic cancer mortality [19, 21, 22, 25, 28]. Our results indicated that the high versus the low plasma 25(OH)D levels was significantly associated with reduced pancreatic cancer mortality without significant heterogeneity (HR=0.81, 95% CI=0.68–0.96, I^2^=43.0%; Figure 2), and the result of Begg's and Egger's tests showed no evidence of publication bias (*P*_Begg's_ = 1.00, *P*_Egger's_ = 0.80, Figure 3). Moreover, we performed a subgroup analysis including only advanced disease, and the result indicated that high plasma 25(OH)D levels tended toward a favorable survival for locally advanced or metastatic disease, although statistical significance was not reached (HR=0.76, 95% CI=0.55–1.04, I^2^=51.4%). Of those five studies, only two studies provided the HR on progression-free survival (PFS) [22, 25], and the pooled HR indicated no association between plasma 25(OH)D levels and PFS for pancreatic cancer (HR=1.06, 95% CI=0.84–1.33, I^2^=12.9%).

### Association between plasma 25(OH)D levels and pancreatic cancer risk

**Figure 4:**
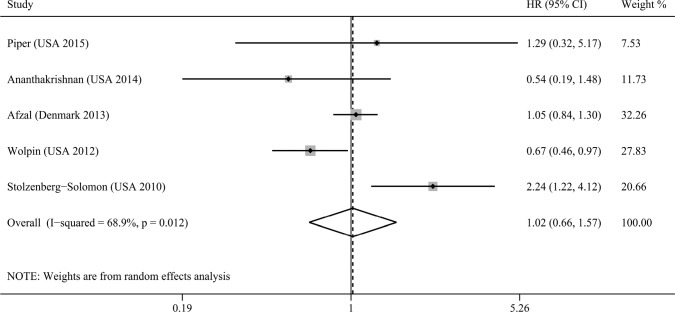
Meta-analysis of the association between plasma 25(OH)D levels and pancreatic cancer risk

There were five studies assessing the association between plasma 25(OH)D levels and pancreatic cancer risk [17, 18, 23, 24, 27]. And comparing with low plasma 25(OH)D levels, high plasma 25(OH)D levels could not reduce pancreatic cancer risk (RR=1.02, 95% CI=0.66–1.57, I^2^=68.9%; Figure 4), without publication bias (*P*_Begg's_ = 1.00, *P*_Egger's_ = 0.94, Figure 3). The sensitivity analysis indicated that the result was not obviously affected by any single study (Supplementary Figure 1). And the Galbraith plot showed that the study by Stolzenberg et al. [24] may contribute substantial heterogeneity (Supplementary Figure 2). Exclusion of the study by Stolzenberg et al. [24] could obtain similar result, with reduced heterogeneity (RR=0.93, 95% CI=0.77–1.11, I^2^=44.9%). Stratifying by sex, smoking status, ethnicity, and time between plasma collection and diagnosis, these subgroup analyses obtained similar results, indicating no association between plasma 25(OH)D levels and pancreatic cancer risk.

### Association between vitamin D intake and pancreatic cancer risk

**Figure 5:**
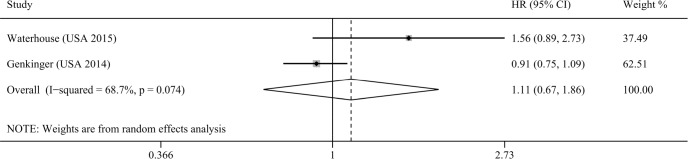
Meta-analysis of the association between vitamin D intake and pancreatic cancer risk

Two studies assessed the association between vitamin D intake and pancreatic cancer risk [20, 26]. Our result indicated that total vitamin D intake was not associated with pancreatic cancer risk (RR=1.11, 95% CI=0.67–1.86, I^2^=68.7%; Figure 5). Subgroup analysis based on dietary vitamin D intake was performed, and similar result was obtained (RR=1.16, 95% CI=0.85–1.59, I^2^=58.2%). However, our study could not assess the impact of supplementary vitamin D intake on pancreatic cancer risk because of the limited number of included studies.

## DISCUSSION

Recent studies have shown that vitamin D status could reduce the incidence and improve the prognosis for numerous solid cancer risks [8–10, 29, 30]. However, there were no general agreements on the association between vitamin D status and pancreatic cancer risk as well as mortality.

Our studies included twelve eligible studies to evaluate the association between vitamin D status and pancreatic cancer risk and mortality. The results indicated that high plasma 25(OH)D levels were significantly associated with reduced pancreatic cancer mortality for pancreatic cancer, but not with PFS. And patients with high plasma 25(OH)D levels had a 19% lower risk for mortality (HR=0.81, 95% CI=0.68–0.96). However, our results indicated that high vitamin D intake or plasma 25(OH)D levels could not reduce the incidence of pancreatic cancer.

Our results were consistent with the 25(OH)D levels-cancer survival relationship reported from several studies, which have shown a favorable effect of vitamin D on cancer survival. Grant et al. reported that lower plasma 25(OH)D levels among African Americans than white Americans may explain many of the cancer survival disparities after consideration of socioeconomic status, stage at time of diagnosis, and treatment [31]. Gaksch et al. analyzed 26916 individuals and the results showed that individuals with low plasma 25(OH)D levels had unfavorable cancer mortality and plasma 25(OH)D levels may affect cancer mortality in a nonlinear relationship [32, 33]. A population-based study by Tretli et al. also showed that higher plasma 25(OH)D levels were positively associated with the survival for cancers of the breast, colon, lung, and lymphoma [34].

Although the intrinsic molecular mechanisms on vitamin D-cancer survival relationship remained unclear, there were some plausible mechanisms. 25(OH)D could be converted to 1,25-dihydroxyvitamin D [1,25(OH)_2_D], the active form of vitamin D, by 25(OH)D-1α-hydroxylase in pancreatic cell and 1,25(OH)_2_D bind to the vitamin D receptor (VDR) [35–37]. Then the 1,25(OH)_2_D-VDR complex promoted transcription of target genes involved in inhibition of proliferation and angiogenesis and induction of apoptosis and differentiation by interacting with vitamin D response elements (VDRE) [12, 38–40]. Moreover, some genes that lacked VDRE in their promoter regions were also transcriptionally affected by 1,25(OH)_2_D, which suggested that 1,25(OH)_2_D may have VDR-independent pathway for the antitumor effects [40]. And several studies have reported that 1,25(OH)_2_D could suppress the expression of epidermal growth factor and insulin-like growth factor 1 and promote the expression of transformation growth factor-β, which was an inhibitory growth factor, through VDR-independent pathway [41, 42]. In addition, pancreatic islet cells expressed 25(OH)D-1α-hydroxylase and VDR [43]. Thus, 25(OH)D may be linked to endocrine pancreatic function and inhibit pancreatic cancer development through regulation of insulin synthesis, binding, and responsiveness [14, 44–46]. Further studies are needed to elucidate the precise antitumor mechanisms, including VDR-dependent and VDR-independent pathways induced by 1,25(OH)_2_D.

Considering the antitumor activity of 1,25(OH)_2_D against pancreatic cancer, several studies were performed to evaluate the therapeutic effect of 1,25(OH)_2_D when used in combination with chemotherapy. A phase II study assessing calcitriol-enhanced docetaxel regimen showed that high-dose calcitriol with docetaxel may have activity in incurable pancreatic cancer, with a modest increase in time to progression when compared to single docetaxel regimen [47]. Barreto et al. also reported that the perioperative vitamin D use was safe in patients with operable pancreatic cancer [48]. Similar result was observed in prostate cancer, and a double-blind randomized phase II study by Beer et al. demonstrated that high-dose calcitriol plus docetaxel could produce 58% prostate-specific antigen decline (>50% confirmed 4weeks later) and was associated with improved overall survival (HR=0.6, 95% CI=0.45–0.97) [49]. The reasons for the synergistic effect of 1,25(OH)_2_D and chemotherapy were unclear. In addition to the above-mentioned mechanisms, vitamin D could markedly reduce inflammation and fibrosis in pancreatic tumor stroma and then induced stromal remodeling and increased intratumoral chemotherapy drug compared to chemotherapy alone because the VDR expressed in pancreatic tumor stroma acted as a master transcriptional regulator of pancreatic stellate cells to reprise the quiescent state [50]. Thus, expression of VDR in cancer cell and stroma cell may be a therapeutic target for pancreatic cancer. Despite the fact that 1,25(OH)_2_D was the biologically active form of vitamin D and directly exerted biological effects in tissue, the clinical use of 1,25(OH)_2_D may be impeded by the lethal side effects of hypercalcemia and hypercalciuria [40]. One strategy was using synthetic 1,25(OH)_2_D analogs in an effort to avoid those side effects. And numerous studies have shown that 1,25(OH)_2_D analogs exhibited comparable antitumor actions [51, 52]. Another strategy was administration of inactive prohormone 25(OH)D. Further studies are needed to evaluate the clinical benefit of vitamin D and its analogs in pancreatic cancer patients and to develop novel vitamin D analogs with better antitumor effects and lower side effects.

Although *in vitro* experimental studies have noted the protective effect of vitamin D and pancreatic cancer [53], both our pooled result and the included studies indicated null protective associations between vitamin D intake and pancreatic cancer risk. The possible explanation was that vitamin D intake was a small fraction of total vitamin D from all sources and meat intake, as an important source of vitamin D intake, was generally not considered in studies [54]. Indeed, studies have shown that vitamin D intake could not appropriately reflect internal vitamin D status because internal vitamin D primarily produced from endogenous synthesis from sunlight exposure [6, 55], and there was no sufficient evidence to support a significantly linear association between vitamin D intake and plasma 25(OH)D levels. Thus, it may be understandable that vitamin D intake was not a good indicator for pancreatic cancer risk. In terms of plasma 25(OH)D levels, several epidemiological studies have shown inconsistent and conflicted results. Wolpin et al. conducted a study assessing the chemopreventive effect of plasma 25(OH)D levels and demonstrated that higher plasma levels of 25(OH)D were associated with a lower risk of pancreatic cancer, consistent with their previous study [27, 56]. In contrast, the Prostate, Lung, Colorectal and Ovarian Screening Trial cohort showed an insignificant association between plasma 25(OH)D levels and pancreatic cancer risk [23, 57], and the Copenhagen City Heart Study also showed a similar result [17]. Surprisingly, however, a prospective nested case-control study in the Alpha-Tocopherol, Beta-arotene Cancer Prevention study of male Finnish smokers showed that high plasma 25(OH)D levels could increase the risk of pancreatic cancer (OR=2.92, 95% CI=1.56–5.48) [58]. The potential hypothesis for why male Finnish smokers with high plasma 25(OH)D levels had an increased pancreatic cancer risk may be that the interrelation between plasma 25(OH)D levels and pancreatic cancer was different for smokers and nonsmokers [59]. Studies suggested that cigarette smoking has repeatedly been associated with reductions in plasma levels of 25(OH)D [60, 61]. And the effects of smoking on vitamin D status may be involved in the carcinogenic effects of tobacco smoking [62]. Large studies are required to determine whether the associations between plasma 25(OH)D levels, vitamin D intake and pancreatic cancer risk are different among groups with different risk profiles.

Vitamin D was primarily derived from UVB radiation of precursors in skin [63], and thus there have been several UVB indices used in epidemiological studies on pancreatic cancer, including solar UVB dose, solar UV dose, solar radiation dose, and latitude [64]. Several studies evaluated the UVB-vitamin D-cancer hypothesis based on geographical variation of pancreatic cancer incidence and/or mortality [63, 65–67]. Grant et al. and Tran et al. showed that solar UVB or UV radiation was inversely associated with pancreatic cancer risk [63, 67]. Kinoshita et al. reported that high solar radiation could reduce pancreatic cancer risk [65]. Similarly, Neale et al. found a significant association between latitude, UV radiation and pancreatic cancer mortality, with a 1.5% decrease in mortality for every 10-kJ/m^2^ increase in yearly UV radiation [66]. Studies suggested that the inverse association between UVB radiation and pancreatic cancer risk and mortality may be mainly mediated by production of vitamin D. However, our present result did not found a significant association between plasma 25(OH)D levels and pancreatic cancer risk. Thus, further studies are needed to explore not only the association between UVB radiation and plasma 25(OH)D levels but also whether there are other pathways contribute to the protective effect of UVB radiation.

Several limitations of our meta-analysis should be considered. First, high plasma 25(OH)D levels may be correlated to favorable health status impacting survival. Non-differential misclassifications of vitamin D status may exist among some included studies, and some inherent confounding factors could not be solved perfectly in included studies. Thus, an underestimation of risk estimates of vitamin D on pancreatic cancer may exist in this study. Second, the included studies on pancreatic cancer risk used a single measure of plasma 25(OH)D levels and had long median follow-up period, ranging from 6.5 to 21 years. Thus, there was obvious concern that the studies with long follow-up period may lead to errors because a single 25(OH)D levels may be inappropriate for long-term vitamin D status and lose predictive ability for pancreatic cancer risk over time. Indeed, several studies have investigated the effect of follow-up period on the association between plasma 25(OH)D levels and cancer incidence and consistently showed that the association decreased with longer follow-up period [68–70]. Therefore, multiple 25(OH)D level measurements would be helpful and yield more accurate results, perhaps every 2 years and in different seasons. Further studies are needed to accurately evaluate the associations between plasma 25(OH)D levels and pancreatic cancer risk through multiple 25(OH)D level measurements during follow-up period. Third, significant heterogeneity existed among the studies, especially in the analysis on plasma 25(OH)D levels and pancreatic cancer risk, and could not be eliminated or explained completely because of differences in patient characteristics (i.e., age, sex and race) or treatment strategies. And we addressed the heterogeneity by using a relatively conservative random-effects model and Galbraith plot analysis. Therefore, the antitumor and therapeutic effect of vitamin D status should be confirmed with further large-scale multicenter studies with homogeneous patients. In addition, only two studies were included in the analysis evaluating vitamin D intake and pancreatic cancer risk; thus, the limited number of studies may impact the statistical power and limit interpretation of the results.

In conclusion, our results indicate that high plasma 25(OH)D levels was significantly associated with favorable survival for pancreatic cancer patients. No significant associations were observed between vitamin D intake or plasma 25(OH)D levels and pancreatic cancer risk. It is should be noted that most studies were confined to USA, which may impact the generalization of present results for pancreatic cancer worldwide. Further large-scale multicenter studies are required to assess the protective effect of vitamin D for pancreatic cancer patients worldwide.

## MATERIALS AND METHODS

### Literature search

A literature search for relevant studies was conducted using PubMed and Embase databases. Besides, a manual search for potential studies was also conducted using the reference lists of reviews and relevant studies. The following search terms were used in the database in a free manner and “MeSH Terms”: “vitamin D”, “25-hydroxy vitamin D”, “25-hydroxyvitamin D”, “25 hydroxy vitamin D”, “25 hydroxyvitamin D”, “1,25-dihydroxy vitamin D”, “1,25-dihydroxyvitamin D”, “1,25 dihydroxy vitamin D”, “1,25 dihydroxyvitamin D”, “25-hydroxyvitamin D3 1-alpha-hydroxylase”, “cholecalciferol”, “calcidiol”, “calcifediol”, “calcitriol”, “hydroxycholecalciferol”, “ergocalciferol”, “pancreatic cancer”, “pancreatic tumor”, “pancreatic neoplasm”, “pancreatic carcinoma”, “pancreas cancer”, “pancreas tumor”, “pancreas neoplasm”, and “pancreas carcinoma”. The detailed search strategy was shown in Supplementary File 1

### Eligibility criteria

Studies that met the following eligible criteria were included: (1) the exposure of interest was vitamin D intake, or plasma 25(OH)D levels; (2) the outcome of interest was pancreatic cancer incidence or mortality; (3) the outcome measures [odds risk (OR), risk ratio (RR) or hazard ratio (HR)] and corresponding 95% confidence interval (CI) could be extracted; and (4) the study design was cohort study or case–control study with at least one of the outcome measures of interest. If several duplicated studies were published from the same population, only the most recent study was included and the data that only reported in excluded duplicated studies were also extracted.

### Data extraction and quality assessment

Two authors reviewed the studies and extracted data independently. The following data was extracted: first author, publication year and country, study design, sample size, number of case, age and sex of patient, smoking status, tumor characteristics, outcome measures with corresponding 95% CIs, and adjusted variables. Outcome measures with the greatest degree of adjustment for potential confounders were extracted. The quality of the included studies was assessed using Newcastle–Ottawa Scale (NOS) criteria [71]. Any disagreements were resolved by discussion.

### Statistical analysis

RR was used as a measure to evaluate the association between exposure and pancreatic cancer risk. OR in a case-control study was used as RR because the pancreatic cancer incidence was sufficiently rare and the OR were close to the RR [72]. HR was used as a measure to evaluate the association between plasma 25(OH)D levels and pancreatic cancer mortality. If HRs and 95% CIs were not reported directly, the values were recalculated from the available data [73]. The overall analysis was performed by evaluating all the relevant studies. Simultaneously, subgroup analysis was performed stratified by sex, tumor characteristics, smoking status, or ethnicity.

The heterogeneity among studies was evaluated using Cochran Q test and the *I*^2^ statistic [74]. A fixed-effects model was used if there was not significant heterogeneity among studies; otherwise, the random-effects model was used. Galbraith plot was used to explore the potential sources of heterogeneity. Publication bias was evaluated using Begg's and Egger's tests [75, 76]. A sensitivity analysis was performed to evaluate the stability of result and influence of a single study using the leave-one-out approach.

All statistical analyses were performed using Stata software 12.0 (Stata Corporation, College Station, TX, USA). A two-sided P value <0.05 was considered statistically significant.

## SUPPLEMENTARY MATERIALS FIGURES



## Abbreviations

1,25(OH)_2_D: 1,25-dihydroxyvitamin D
25(OH)D: 25-hydroxyvitamin D
CI: confidence interval
HR: hazard ratio
PFS: progression-free survival
RR: risk ratio
VDR: vitamin D receptor
VDRE: vitamin D response element
