# Supplementary material for: Plasma 25-hydroxyvitamin D levels, vitamin D intake, and pancreatic cancer risk or mortality: a meta-analysis
Source: Oncotarget. 2017 Jun 29;8(38):64395–406. doi: 10.18632/oncotarget.18888 (PMC5610011; doi:10.18632/oncotarget.18888)
Supplement: Supplementary file 1 [file oncotarget-08-64395-s001.pdf]

Plasma 25-hydroxyvitamin D levels, vitamin D intake, and pancreatic cancer risk or mortality: a meta-analysis

SUPPLEMENTARY MATERIALS

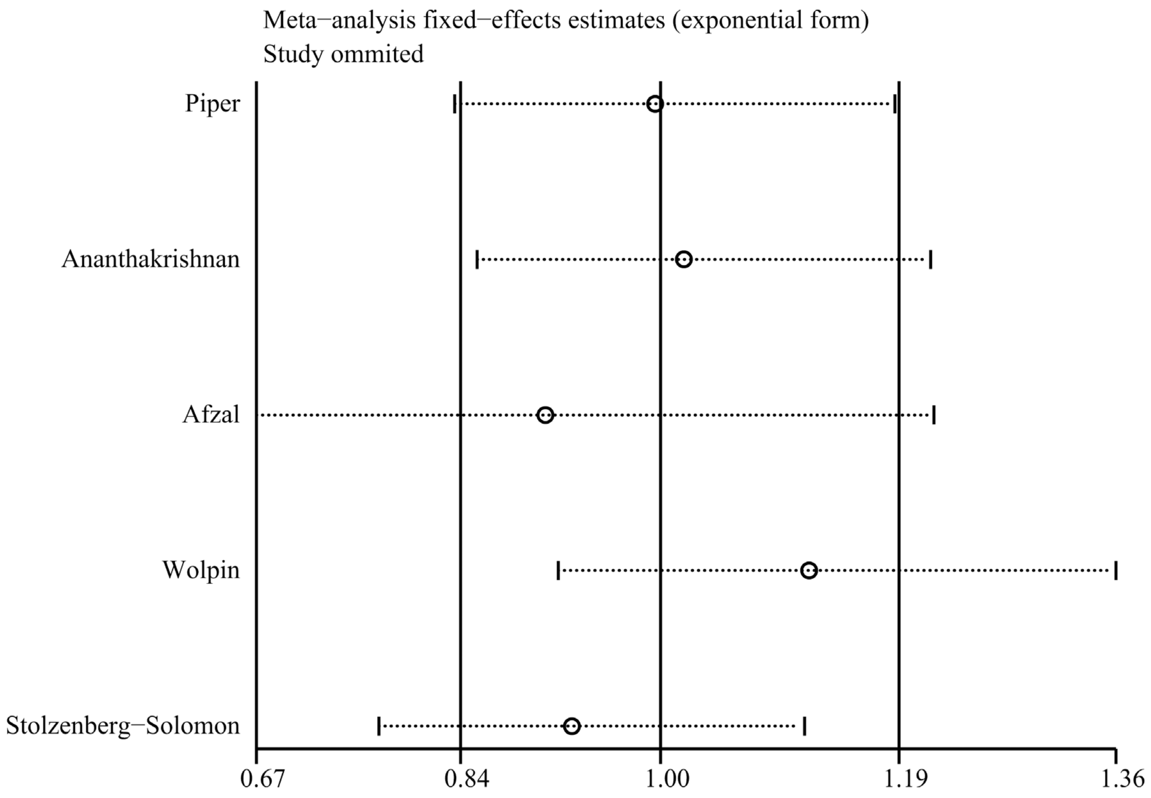

Supplementary Figure 1: Sensitivity analysis on plasma 25(OH)D levels and pancreatic cancer risk based on leave-one-out approach.

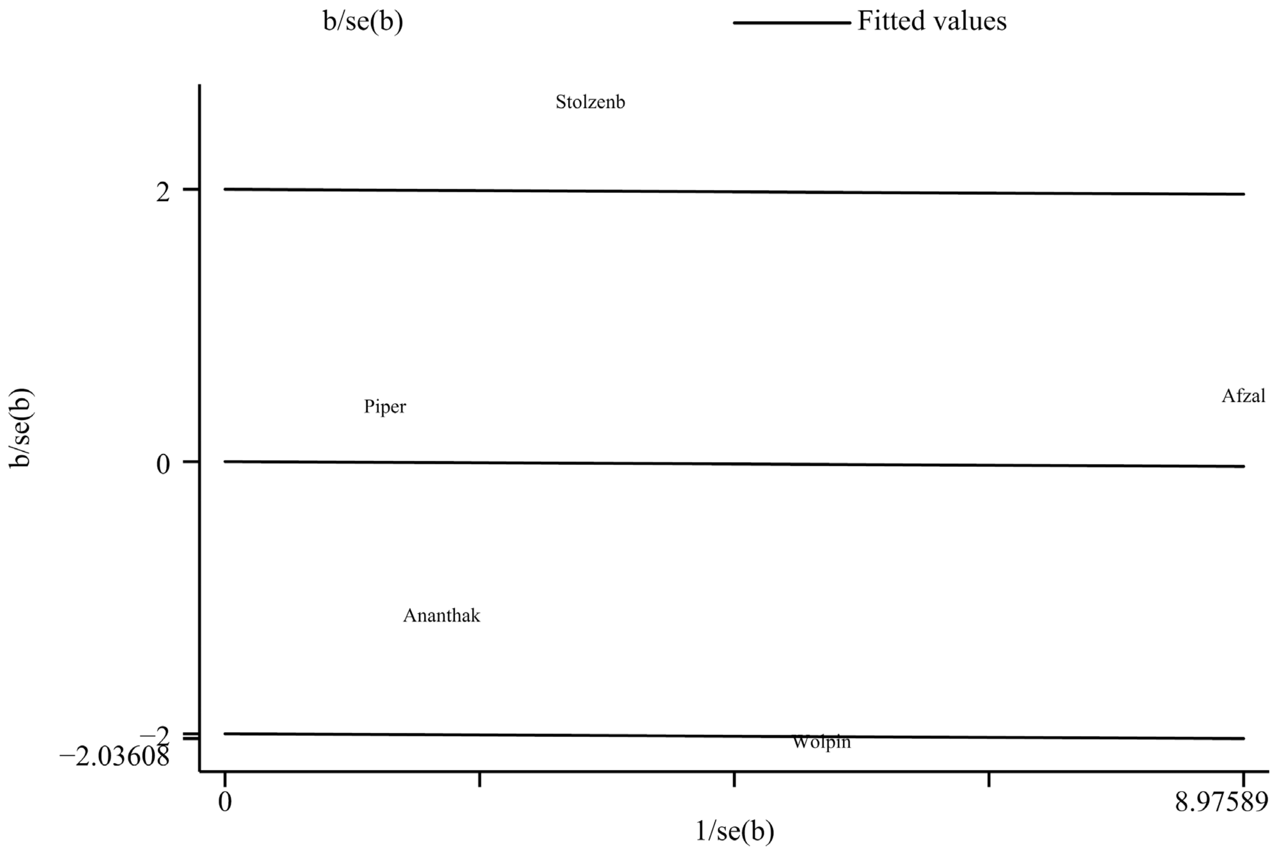

Supplementary Figure 2: The result of Galbraith plot.

Supplementary File 1: The detailed search strategy in the databases

(("vitamin d"[MeSH Terms] OR "vitamin d"[All Fields] OR “ergocalciferols”[MeSH Terms] OR “ergocalciferols”[All Fields]) OR (25-hydroxy[All Fields] AND ("vitamin d"[MeSH Terms] OR "vitamin d"[All Fields] OR “ergocalciferols”[MeSH Terms] OR “ergocalciferols”[All Fields])) OR ("25-hydroxyvitamin D"[Supplementary Concept] OR "25-hydroxyvitamin D"[All Fields] OR "25 hydroxyvitamin d"[All Fields] OR “calcifediol”[MeSH Terms] OR “calcifediol”[All Fields]) OR (25[All Fields] AND hydroxy[All Fields] AND ("vitamin d"[MeSH Terms] OR "vitamin d"[All Fields] OR “ergocalciferols”[MeSH Terms] OR “ergocalciferols”[All Fields])) OR ("25-hydroxyvitamin D"[Supplementary Concept] OR "25-hydroxyvitamin D"[All Fields] OR "25 hydroxyvitamin d"[All Fields] OR “calcifediol”[MeSH Terms] OR “calcifediol”[All Fields]) OR (1[All Fields] AND 25-dihydroxy[All Fields] AND ("vitamin d"[MeSH Terms] OR "vitamin d"[All Fields] OR “ergocalciferols”[MeSH Terms] OR “ergocalciferols”[All Fields])) OR ("1,25-dihydroxyvitamin D"[Supplementary Concept] OR "1,25-dihydroxyvitamin D"[All Fields] OR "1,25 dihydroxyvitamin d"[All Fields]) OR (1[All Fields] AND 25[All Fields] AND dihydroxy[All Fields] AND ("vitamin d"[MeSH Terms] OR "vitamin d"[All Fields] OR “ergocalciferols”[MeSH Terms] OR “ergocalciferols”[All Fields])) OR ("1,25-dihydroxyvitamin D"[Supplementary Concept] OR "1,25-dihydroxyvitamin D"[All Fields] OR "1,25 dihydroxyvitamin d"[All Fields]) OR ("25-hydroxyvitamin d3 1-alpha-hydroxylase"[MeSH Terms] OR ("25-hydroxyvitamin"[All Fields] AND "d3"[All Fields] AND "1-alpha-hydroxylase"[All Fields]) OR "25-hydroxyvitamin d3 1-alpha-hydroxylase"[All Fields] OR "25 hydroxyvitamin d3 1 alpha hydroxylase"[All Fields]) OR (“cholecalciferol”[MeSH Terms] OR “cholecalciferol”[All Fields]) OR (“calcifediol”[MeSH Terms] OR “calcifediol”[All Fields] OR “calcidiol”[All Fields]) OR (“calcifediol”[MeSH Terms] OR “calcifediol”[All Fields]) OR (“calcitriol”[MeSH Terms] OR “calcitriol”[All Fields]) OR (“hydroxycholecalciferols”[MeSH Terms] OR “hydroxycholecalciferols”[All Fields] OR “hydroxycholecalciferol”[All Fields]) OR (“ergocalciferols”[MeSH Terms] OR “ergocalciferols”[All Fields] OR “ergocalciferol”[All Fields])) AND (("pancreatic neoplasms"[MeSH Terms] OR (“pancreatic”[All Fields] AND “neoplasms”[All Fields]) OR "pancreatic neoplasms"[All Fields] OR (“pancreatic”[All Fields] AND “cancer”[All Fields]) OR "pancreatic cancer"[All Fields]) OR ("pancreatic neoplasms"[MeSH Terms] OR (“pancreatic”[All Fields] AND “neoplasms”[All Fields]) OR "pancreatic neoplasms"[All Fields] OR (“pancreatic”[All Fields] AND “tumor”[All Fields]) OR "pancreatic tumor"[All Fields]) OR ("pancreatic neoplasms"[MeSH Terms] OR (“pancreatic”[All Fields] AND “neoplasms”[All Fields]) OR "pancreatic neoplasms"[All Fields] OR (“pancreatic”[All Fields] AND “neoplasm”[All Fields]) OR "pancreatic neoplasm"[All Fields]) OR ("Pancreatic Carcinoma"[Supplementary Concept] OR "Pancreatic Carcinoma"[All Fields] OR "pancreatic carcinoma"[All Fields]) OR ("pancreatic neoplasms"[MeSH Terms] OR (“pancreatic”[All Fields] AND “neoplasms”[All Fields]) OR "pancreatic neoplasms"[All Fields] OR (“pancreas”[All Fields] AND “cancer”[All Fields]) OR "pancreas cancer"[All Fields]) OR ("pancreatic neoplasms"[MeSH Terms] OR (“pancreatic”[All Fields] AND “neoplasms”[All Fields]) OR "pancreatic neoplasms"[All Fields] OR (“pancreas”[All Fields] AND “tumor”[All Fields]) OR "pancreas tumor"[All Fields]) OR ("pancreatic neoplasms"[MeSH Terms] OR (“pancreatic”[All Fields] AND “neoplasms”[All Fields]) OR "pancreatic neoplasms"[All Fields] OR (“pancreas”[All Fields] AND “neoplasm”[All Fields]) OR "pancreas neoplasm"[All Fields]) OR ((“pancreas”[MeSH Terms] OR “pancreas”[All Fields]) AND (“carcinoma”[MeSH Terms] OR “carcinoma”[All Fields])))
